# Prognostic value of CMR in suspected CHD: 3 year follow-up of the CE-MARC study

**DOI:** 10.1186/1532-429X-16-S1-O36

**Published:** 2014-01-16

**Authors:** John P Greenwood, Bernhard A Herzog, Julia Brown, Colin C Everett, Jane Nixon, Petra Bijsterveld, Neil Maredia, Manish Motwani, Stephen G Ball, Sven Plein

**Affiliations:** 1 Cardiology, Leeds General Infirmary, Leeds, UK; 2 Division of Cardiovascular and Diabetes Research, University of Leeds, Leeds, UK; 3 Clinical Trials Research Unit, University of Leeds, Leeds, UK

## Background

There are limited prospective, prognostic data for cardiovascular magnetic resonance (CMR) in the same patients with suspected coronary heart disease (CHD). CE-MARC established the diagnostic performance of CMR (and SPECT), with a predefined objective to determine both modalities' ability to predict major adverse cardiovascular events (MACE).

## Methods

All CE-MARC patients underwent annual follow-up for 3 years to assess the occurrence of MACE (cardiovascular death, acute coronary syndrome, unscheduled revascularization or hospital admission for any cardiovascular cause). Time to MACE was assessed by univariate (log-rank test) and multivariate (Cox proportional hazards regression) analysis after adjustment for major cardiovascular risk factors. Net reclassification improvement (NRI) and discrimination (IDI) of the risk of MACE with CMR, incremental to standard clinical risk factors was calculated.

## Results

**Figure 1:**
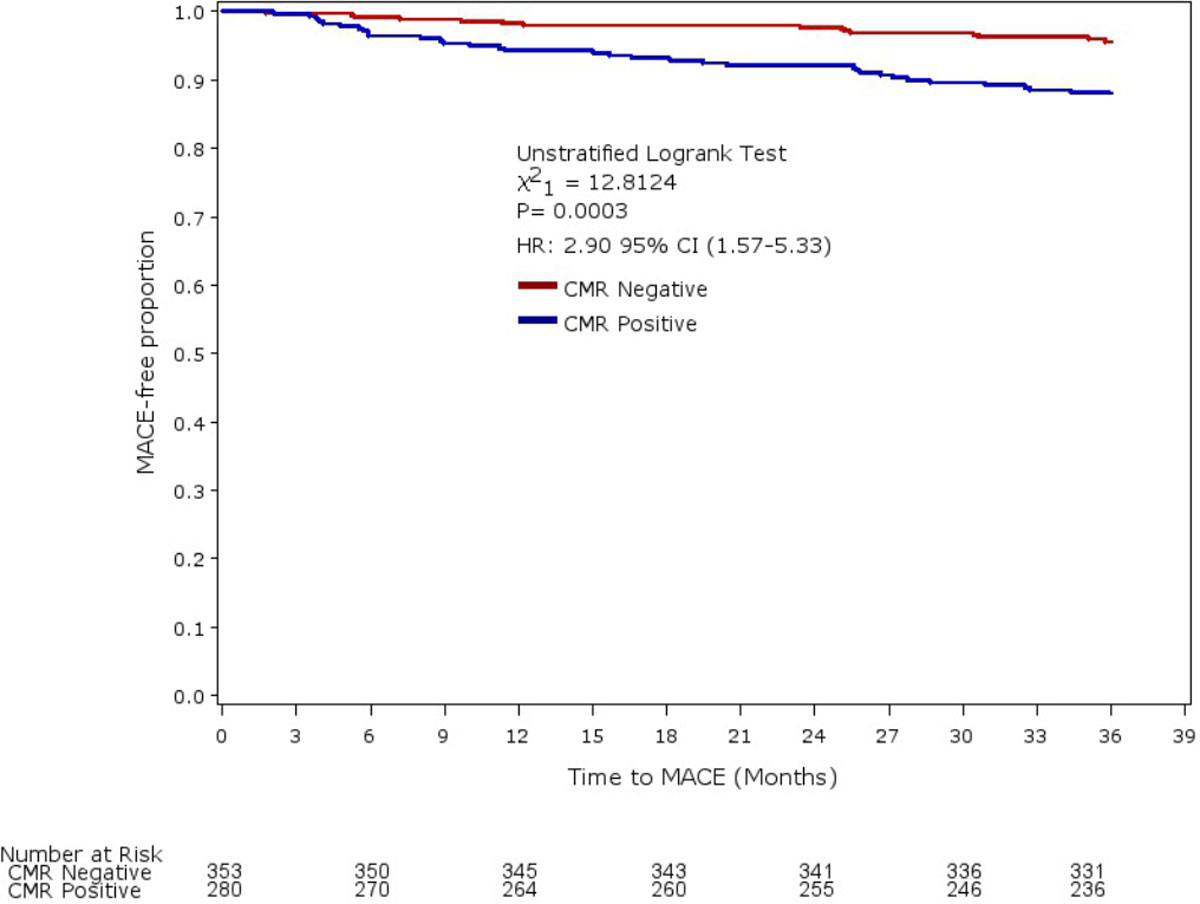
**Kaplan-Meier event curves for CMR for MACE**.

747(99.3%) of 752 patients recruited had complete follow-up. Of 633 who underwent both CMR and SPECT, 41(6.5%) had at least 1 MACE event. Abnormal CMR (HR 2.51; 95%CI, 1.32-4.79; p = 0.005) findings were strong and independent predictors of MACE (Figure 1). CMR also showed improved reclassification and risk stratification (Net Reclassification Index, NRI 0.57(95%CI 0.26-0.89); Integrated Discrimination Improvement, IDI 0.02 (95%CI 0.002-0.06)).

## Conclusions

Three-year follow-up of the CE-MARC trial population demonstrates that abnormal CMR findings are independent predictors of MACE, and can reclassify patient risk beyond the standard major cardiovascular risk factors. This further supports the role of CMR for the assessment and management of patients with suspected CHD.

## Funding

British Heart Foundation (RG/05/004).

